# Syphilis detection rate trend in aged people: Brazil, 2011–2019

**DOI:** 10.1590/1980-549720230033

**Published:** 2023-07-10

**Authors:** Zildânya da Silva Barros, Bruna Grazielle Mendes Rodrigues, Karoline de Macêdo Gonçalves Frota, Jardeliny Corrêa da Penha, Fernando Ferraz do Nascimento, Malvina Thais Pacheco Rodrigues, Márcio Dênis Medeiros Mascarenhas

**Affiliations:** I Universidade Federal do Piauí – Teresina (PI), Brazil.

**Keywords:** Syphilis, Aged, Health information systems, Time series studies, Sífilis, Idoso, Sistemas de informação em saúde, Estudos de séries temporais

## Abstract

**Objective:**

To analyze the trend in the detection rate of syphilis in elderly people in Brazil from 2011 to 2019.

**Methods:**

An ecological, time-series study with data from the Notifiable Diseases Information System. The temporal trend of syphilis detection rates was analyzed according to the Prais-Winsten linear regression method.

**Results:**

62,765 cases of syphilis in aged people were reported. There was a growing trend in the rate of syphilis detection in aged people in Brazil. The increase was of approximately six times, with a mean increase of 25% each year (annual percent change [APC]: 25.0; 95%CI 22.1–28.1). The increase in the detection rate was identified in both genders and for all age groups, with emphasis on the increase in females (APC: 49.1; 95%CI 21.9–26.8) and in the group aged 70 to 79 years old (APC: 25.8; 95%CI 23.3–28.3). All macro-regions of the country showed an increasing trend, with emphasis on the Northeast (APC: 51.2; 95%CI 43.0–59.8) and South (APC: 49.2; 95%CI 32.3–68.3).

**Conclusion:**

The growing trend in the detection rate of syphilis in aged people throughout Brazil highlights the need for planning and developing effective and multidisciplinary prevention actions and assistance adapted to this public.

## RESUMO

**Objetivo:**

Analisar a tendência da taxa de detecção de sífilis em pessoas idosas no Brasil no período de 2011 a 2019.

**Métodos:**

Estudo ecológico, de série temporal, com dados do Sistema de Informação de Agravos de Notificação. Analisou-se a tendência temporal das taxas de detecção de sífilis segundo o método de regressão linear de Prais-Winsten.

**Resultados:**

Foram notificados 62.765 casos de sífilis em pessoas idosas. Verificou-se tendência crescente na taxa de detecção de sífilis em pessoas idosas no Brasil. O aumento foi de, aproximadamente, seis vezes, com incremento médio de 25% a cada ano (variação percentual anual [VPA]: 25,0; IC95% 22,1–28,1). O aumento na taxa de detecção foi identificado em ambos os sexos e para todos os grupos etários, com destaque para o incremento no sexo feminino (VPA: 49,1; IC95% 21,9–26,8) e no grupo com 70 a 79 anos de idade (VPA: 25,8; IC95% 23,3–28,3). Todas as macrorregiões do país apresentaram tendência crescente, com destaque para as regiões Nordeste (VPA: 51,2; IC95% 43,0–59,8) e Sul (VPA: 49,2; IC95% 32,3–68,3).

**Conclusão:**

A tendência crescente da taxa de detecção de sífilis em pessoas idosas em todo território brasileiro evidencia a necessidade de planejamento e desenvolvimento de ações efetivas e multiprofissionais de prevenção e assistência adaptada a esse público.

## INTRODUCTION

Brazil has shown exponential growth in the population of aged people. It is estimated that, by 2050, about half of the Brazilian population will be consisted of people aged 60 years old and older, reaching a number equal to or greater than that of children and young people from zero to 15 years of age^1^.

New technologies in the health area, advances in medication and hormone replacement, improvement in the levels of personal, environmental, and food hygiene may have contributed to the increase in the aged population and, consequently, to the extension of sexual life at an older age^2^, even after the age of 80^3^.

Low adherence to the use of condoms during sexual intercourse can turn the aged into targets of sexually transmitted infections (STIs), including syphilis. In addition, other factors contribute to the greater vulnerability of the aged to STIs: impaired immunity, poor knowledge about STIs and prevention methods, prejudice, shame and myths about the use of condoms, as well as cultural barriers on sexuality^4^.

Studies indicate that North America^5^, the United Kingdom^6^, Korea^7^, and Australia^8^ showed a significant increase in cases of syphilis in individuals over 50 years of age, with associated factors such as marital status, lack of information, non-use of condoms and increased life expectancy^5–8^.

This increase was also observed in the Brazilian territory. In the period from 2010 to 2021, 168,871 cases of syphilis were registered in people over 50 years of age, representing about 18.4% of cases^9^. This STI is responsible for severe outcomes, including genital ulcers and chronic damage to the nervous and cardiovascular systems, bones and mucous tissues^10,11^. In addition, for the aged, syphilis can lead to dementia or death, as this group has the most fragile health^12^.

Considering that the treatment recommended by the Ministry of Health is through the application of penicillin G benzathine and that this drug, between 2014 and 2017, had its stock reduced in health services, being restricted for use in cases of congenital syphilis and in pregnant women, brings up the issue of greater attention to such types of syphilis^13,14^.

It should also be noted that most scientific studies are focused on acquired syphilis in adults, gestational and congenital syphilis, as they are more common and because of the damage to the fetus. Aged individuals are generally considered as people who do not have sex^15^, and thus end up not being the focus of studies.

In view of this, it is important to know the Brazilian reality, especially the occurrence of this condition in aged people, so that, with the results, new public policies for this population are created and improved and strategies for the prevention and control of this disease are effectively implemented. Thus, the study aimed to analyze the trend in the detection rate of syphilis in aged people in Brazil, from 2011 to 2019.

## METHODS

This is an ecological, time-series study on notifications of syphilis cases in aged people (≥60 years old) in Brazil, from 2011 to 2019. The following units of analysis were adopted: states, the Federal District, and the five geographic macro-regions of Brazil (North, Northeast, Southeast, South, and Center-West).

Acquired syphilis was included in the National List of Compulsorily Notifiable Diseases and Injuries (*Lista Nacional de Doenças e Agravos de Notificação Compulsória* – LNDANC) in 2010, through Ordinance No. 2.472^16^. A case of acquired syphilis is defined as “every asymptomatic individual or with clinical evidence of primary or secondary syphilis (presence of hard chancre or lesions compatible with secondary syphilis), with a reactive non-treponemal test with any titration and a reactive treponemal test”^17^. Upon diagnosis, cases of syphilis are notified by completing a notification form and registered in the Brazilian Notifiable Diseases Information System (*Sistema de Informação de Agravos de Notificação* – SINAN).

In this study, data referring to notifications of syphilis in people aged 60 years old and older contained in SINAN and population data made available by the Brazilian Institute of Geography and Statistics (*Instituto Brasileiro de Geografia e Estatística* – IBGE) were obtained by TabNet, an electronic tabulator available on the website of the Department of the Brazilian Unified Health System (*Departamento de Informática do Sistema Único de Saúde* – DATASUS) (available at: http://www.datasus.gov.br; cited on Aug 20, 2022).

The study period began in 2011, corresponding to the first year after the disease was included in the LNDANC, and ended in 2019, the first year before the new coronavirus (COVID-19) pandemic, since the restriction of care in health services could have influenced the reduction in notification of diseases and injuries in 2020 and 2021.

Descriptive variables were: gender (female, male), age range (up to 19 years old, 20–59 years old, 60–69, 70–79, 80 years old or older), race (white, black, other), education (illiterate, incomplete elementary school, elementary school, high school, and higher education), state, and geographical macro-regions of residence (North, Northeast, Southeast, South, Center-West).

The detection rate of syphilis cases in aged people was calculated by dividing the number of syphilis cases notified, in a given location, year, gender, and age group, by the population of aged people, in the same location, period, gender and age group. age group, multiplying the result by 100,000.

The temporal trend of syphilis detection rate in the aged was obtained using Prais-Winsten linear regression, which allows obtaining the annual percent change (APC) and the respective 95% confidence intervals (95%CI). In the linear regression, the syphilis detection rate was considered the dependent variable (X), and the year of diagnosis, the independent variable (Y).

For each state, region and country, the model represented by formula I was adopted: *log* (*y_t_*) = *β*_0_ + *β*_1_ *x_t_* + ε_t_^18^. In formula I, *y_t_* represents the syphilis detection rate in the aged in year *t*, *x_t_* represents the year in which the detection rate occurred, and *ε_t_* is the error at time *t*. The absolute value of syphilis detection rate in the aged was converted into the natural logarithm of the rate^18^.

Once the model was adjusted, for each state, region and country, the estimate of *ε*_1_ was obtained, as well as its 95%CI. After this step, APC was obtained using formula II: *VPA* = (10^β^ − 1) × 100.

The 95% confidence interval for APC was also obtained through formula II, replacing the value of *β*_1_ by the respective values of the lower and upper limits of the 95%CI for *β*_1_. Finally, the syphilis detection rate in aged people was classified as increasing (regression coefficient was positive and p<0.05), decreasing (regression coefficient was negative and p<0.05) or stationary (p>0.05). Unlike the joinpoint technique, the Prais-Winsten technique does not identify inflection points in the time series.

The Microsoft Excel 2016 program for Windows was used in the database organization stage and calculation of the syphilis detection rate in the aged. Other statistical analyzes were performed using the Stata software, version 17.0.

This study was not submitted for registration and evaluation by the Research Ethics Committee because it is an analysis of secondary anonymous data available on a public access platform, in accordance with Resolution No. 510, of 2016, of the National Health Council.

## RESULTS

A total of 62,765 cases of syphilis were reported in aged people (≥60 years of age) across Brazil, from 2011 to 2019.

There was a growing trend in the rate of syphilis detection in aged people in Brazil. The increase was approximately sixfold, with a mean increase of 25% each year (APC: 25.0; 95%CI 22.1–28.1). The increase in the detection rate was identified in both genders and for all age groups, with emphasis on females (APC: 49.1; 95%CI 21.9–26.8) and in the group aged 70 to 79 years old (APC: 25.8; 95%CI 23.3–28.3) (Table 1).

**Table 1:** Annual percent change and trend in the crude detection rate of syphilis in the aged (per 100,000 inhabitants), by gender and age group. Brazil, 2011-2019.

Characteristics		Detection rate		APC (%)	95%CI	p-value	Trend
		2011	2019				
Gender							
	Female	6.00	32.82	49.1	21.9–26.8	<0.001	Growing
	Male	10.32	62.10	25.5	22.1–29.0	<0.001	Growing
Age range (in years)							
	60–69	9.45	53.76	24.7	21.5–28.0	<0.001	Growing
	70–79	6.76	40.99	25.8	23.3–28.3	<0.001	Growing
	≥80	4.44	25.38	25.1	22.0–28.2	<0.001	Growing
	Brazil	7.92	45.80	25.0	22.1–28.1	<0.001	Growing

Source: Sistema de Informação de Agravos de Notificação (SINAN). APC: annual percent change; 95%CI: 95% confidence interval.

**Figure 1:**
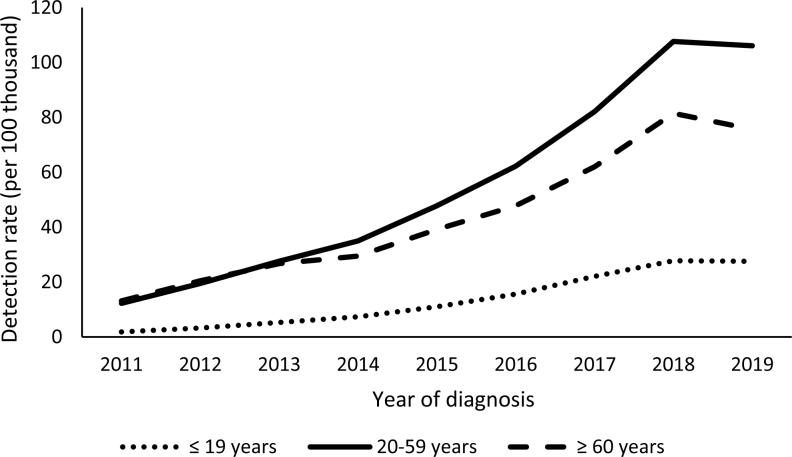
Syphilis detection rate in the general population (per 100,000 inhabitants), according to age range and year of diagnosis. Brazil, 2011–2019.

Source: Sistema de Informação de Agravos de Notificação (SINAN).

Figure 1 shows the evolution of the syphilis detection rate in the general population in Brazil from 2011 to 2019. There was a progressive increase in all stages of life (children and adolescents, adults, and the aged). Until 2013, detection rates among adults and the aged were similar. As of 2014, even with a simultaneous increase, the detection rate in adults surpassed the rate observed in the group of aged people. The highest rate was observed in adults, in 2018 (107.67/100,000 inhabitants), followed by the rate in the aged population (81.48/100,000 inhabitants). The rate in people under 20 years of age was 27.77/100,000 inhabitants.

**Figure 2:**
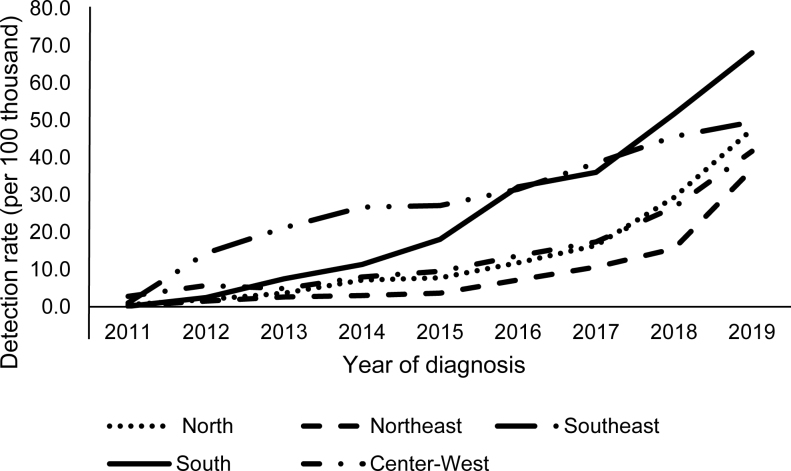
Syphilis detection rate in aged people (per 100,000 inhabitants), according to geographic macro-region and year of diagnosis. Brazil, 2011–2019.

Source: Sistema de Informação de Agravos de Notificação (SINAN).

Figure 2 shows the evolution of the syphilis detection rate in aged people according to geographic macro-region and year of diagnosis. Until 2015, the Southeast Region had the highest detection rate, being surpassed by the South Region, which had the highest detection rate in 2019 (68.1/100,000 inhabitants). The Northeast Region had the lowest detection rate throughout the analyzed period, reaching 36.8/100,000 inhabitants in 2019, following the growth observed for all macro-regions.

The syphilis detection rate in aged people showed an increasing trend (APC: 25.0; 95%CI 22.0–28.1) for the entire Brazilian territory. All macro-regions in the country showed an increasing trend, with emphasis on the Northeast (APC: 51.2; 95%CI 43.0–59.8) and South (APC: 49.2; 95%CI 32.3–68.3), which had the highest annual increments. In 2019, the highest detection rates were recorded in Roraima (99.42/100,000 inhabitants), Paraná (91.80/100,000 inhabitants), Amazonas (84.12/100,000 inhabitants), and the lowest ones in Alagoas (7.55/100,000 inhabitants), Acre (16.11/100,000 inhabitants), Ceará (16.55/100,000 inhabitants). The states with the highest annual increase in the detection rate of syphilis in the aged were Roraima (APC: 97; 95%CI 65.1–135.0), Maranhão (APC: 83.1; 95%CI 58.7–111.2), Amapá (APC: 82.5; 95%CI 56.8–79.7), and Pará (APC: 67.9; 95%CI 56.8–79.7). São Paulo (APC: 10.8; 95%CI 4.6–17.4), Mato Grosso (APC: 18.2; 95%CI 9.1–28.0), Espírito Santo (APC: 18.8; 95%CI % 11.0–27.2), and Ceará (APC: 23.0; 95%CI 1.2–49.6) had the lowest annual increases (Table 2).

**Table 2:** Annual percent change and trend in the crude detection rate of syphilis in the aged (per 100,000 inhabitants), by region and state, Brazil, 2011–2019.

Region/State	Detection rate		APC (%)	95%CI	p-value	Trend
	2011	2019				
North	1.94	45.80	49.1	42.2–56.3	<0.001	Growing
Rondônia*	0.00	37.67	74.9	34.6–127.3	<0.001	Growing
Acre*	0.00	16.11	53.2	32.3–77.3	<0.001	Growing
Amazonas	6.33	84.12	31.1	16.2–47.9	0.001	Growing
Roraima*	0.00	99.42	97.0	65.1–135.0	<0.001	Growing
Pará	0.53	28.41	77.8	56.8–79.7	<0.001	Growing
Amapá*	0.00	68.75	82.5	47.7–125.5	<0.001	Growing
Tocantins*	0.00	53.35	64.1	52.8–76.2	<0.001	Growing
Northeast	1.50	32.81	51.2	43.0–59.8	<0.001	Growing
Maranhão	0.17	25.06	83.1	58.7–111.2	<0.001	Growing
Piauí	0.58	23.82	57.6	46.2–69.8	<0.001	Growing
Ceará	3.71	16.55	23.0	1.2–49.6	0.041	Growing
Rio Grande do Norte	3.09	25.36	28.2	9.1–50.7	0.008	Growing
Paraíba	0.65	23.24	62.6	51.2–74.8	<0.001	Growing
Pernambuco	1.95	60.92	63.0	40.6–88.9	<0.001	Growing
Alagoas	0.35	7.55	62.5	29.9–103.3	0.001	Growing
Sergipe	1.03	18.57	28.8	1.8–62.9	0.039	Growing
Bahia	0.73	40.85	65.5	55.0–76.7	<0.001	Growing
Southeast	14.39	44.76	15.2	10.7–20.0	<0.001	Growing
Minas Gerais	2.34	34.29	43.5	37.0–50.2	<0.001	Growing
Espírito Santo	13.44	61.91	18.8	11.0–27.2	0.001	Growing
Rio de Janeiro	3.25	23.09	30.6	21.4–40.5	<0.001	Growing
São Paulo	25.11	57.31	10.8	4.6–17.4	0.004	Growing
South	2.48	69.92	49.2	32.3–68.3	<0.001	Growing
Paraná	1.86	91.80	62.6	47.9–78.8	<0.001	Growing
Santa Catarina	1.00	56.46	62.6	35.0–95.9	<0.001	Growing
Rio Grande do Sul	3.64	58.61	38.3	20.4–58.8	0.001	Growing
Center-West	5.72	40.09	34.7	30.2–39.3	<0.001	Growing
Mato Grosso do Sul	22.98	59.48	18.2	9.1–28.0	0.002	Growing
Mato Grosso	1.18	32.52	50.7	39.9–62.3	<0.001	Growing
Goiás	1.18	32.04	56.5	50.6–62.6	<0.001	Growing
Federal District	3.30	48.38	59.8	34.3–90.2	<0.001	Growing
Brazil	7.92	45.80	25.0	22.1–28.1	<0.001	Growing

Source: Sistema de Informação de Agravos de Notificação (SINAN).

APC: annual percent change; 95%: 95% confidence interval.

* To calculate the APC, log=1 was considered for the states of Rondônia, Acre, Amapá, Roraima, and Tocantins in the year 2011.

## DISCUSSION

The study identified a growing trend in the rate of detection of syphilis cases in aged people in Brazil, from 2011 to 2019. This trend was also observed in both genders, age groups, states, and geographic regions analyzed in the study. The trends observed for females, the group aged 70 to 79 years old, living in the Northeast and South regions and in the states of Roraima, Maranhão, Amapá, and Pará stood out.

Although the diagnosis and treatment are fast and effective, syphilis affects about 12 million people over 19 years of age around the world, being a great challenge and a serious public health problem^13^. The high incidence of the disease in several countries may be related to the fragility of health services and the low investment of both financial and information resources^19–21^.

In Brazil, syphilis continues to affect mostly people under 59 years of age, but there is a progressive increase among the aged. This fact is also evidenced in a study in China, in which, of the 71,055 cases reported in 2019, 48.8% were of people over 50 years of age^11^.

The occurrence of the disease differs between the genders. Although the highest increase was observed in females, the highest detection rate was observed in males, which may be related to the use of drugs for erectile dysfunction, favoring the maintenance of sexual activity at older ages^22,23^. In addition, men are more exposed to infections due to the greater number of sexual partners throughout their lives, associated with the lack of condom use, in addition to social, environmental, and lifestyle factors^24^.

With regard to age group, there was a decline with advancing age, that is, as age increases, syphilis detection rates decrease. This finding was also observed when analyzing aged people in Cascavel, Paraná, between 2013 and 2016, among whom advancing age results in physiological changes and, consequently, in the reduction of sexual activity^2^.

The increase in the syphilis detection rate was also observed according to the geographic regions of Brazil. The South Region had the highest syphilis detection rate values. Although the population of this region may have better access to health services, in addition to better prepared care and surveillance teams, the increase in detection rates demonstrates the fragility of prevention programs for this pathology, despite the existence of free treatment and preventive methods^25^.

Other relevant factors for the growing trend of syphilis in aged people would be the physiological, psychological, and affective changes in current days, with a preference toward remaining active in different activities, including sex. Allied to this, several improvements for sexual dysfunctions are disseminated, such as drugs for erectile disorders and hormone replacement^26^.

In view of this, adaptations and updates are necessary for the reach and well-being of this phase of life, among them, the breadth of the concept of sexuality, which is not related to the reproductive function, but rather as a source of pleasure and self-esteem. In Brazil, 61.6% of the aged are sexually active, of these, 58.9% have a steady partner, who, for the most part, neglect the use of condoms, thus becoming more vulnerable to the transmission of syphilis^27^.

The increase in the population of this age group should also be noted as a predictor for this evolution, which may reach, in 2025, 32 million Brazilian individuals over 59 years of age^28^, as well as a greater dissemination of the use of rapid tests to diagnose STIs and an improvement in the notification system^13^.

As limitations of the study, there is the lack of detection rates in all years of the historical series given the absence of data in the initial years of mandatory reporting of syphilis cases, in addition to possible underreporting with SINAN. Many aged people do not know how to identify the signs and symptoms of the disease or are ashamed and/or afraid of being judged when seeking medical care, which contributes to the underreporting of cases.

The growing trend in the rate of syphilis detection in aged people throughout Brazil highlights the need for planning and developing effective and multidisciplinary prevention actions and assistance adapted to this public.
